# Cytoskeletal stability and metabolic alterations in primary human macrophages in long-term microgravity

**DOI:** 10.1371/journal.pone.0175599

**Published:** 2017-04-18

**Authors:** Svantje Tauber, Beatrice A. Lauber, Katrin Paulsen, Liliana E. Layer, Martin Lehmann, Swantje Hauschild, Naomi R. Shepherd, Jennifer Polzer, Jürgen Segerer, Cora S. Thiel, Oliver Ullrich

**Affiliations:** 1 Institute of Anatomy, Faculty of Medicine, University of Zurich, Winterthurerstrasse 190, Zurich, Switzerland; 2 Department of Machine Design, Engineering Design and Product Development, Institute of Mechanical Engineering, Otto-von-Guericke-University Magdeburg, Universitätsplatz 2, Magdeburg, Germany; 3 Biozentrum der LMU München, Deptartment of Biology I–Botany, Grosshaderner Strasse 2–4, Planegg-Martinsried, Germany; 4 Airbus Defense and Space, GmbH, Claude-Dornier-Strasse, Immenstaad, Germany; 5 Zurich Center for Integrative Human Physiology (ZIHP), University of Zurich, Winterthurerstrasse 190, Zurich, Switzerland; 6 Space Life Sciences Laboratory (SLSL), Kennedy Space Center, 505 Odyssey Way, Exploration Park, Florida, United States of America; Charles P. Darby Children's Research Institute, 173 Ashley Avenue, Charleston, SC 29425, UNITED STATES

## Abstract

The immune system is one of the most affected systems of the human body during space flight. The cells of the immune system are exceptionally sensitive to microgravity. Thus, serious concerns arise, whether space flight associated weakening of the immune system ultimately precludes the expansion of human presence beyond the Earth's orbit. For human space flight, it is an urgent need to understand the cellular and molecular mechanisms by which altered gravity influences and changes the functions of immune cells. The CELLBOX-PRIME (= CellBox-Primary Human Macrophages in Microgravity Environment) experiment investigated for the first time microgravity-associated long-term alterations in primary human macrophages, one of the most important effector cells of the immune system. The experiment was conducted in the U.S. National Laboratory on board of the International Space Station ISS using the NanoRacks laboratory and Biorack type I standard CELLBOX EUE type IV containers. Upload and download were performed with the SpaceX CRS-3 and the Dragon spaceship on April 18th, 2014 / May 18th, 2014. Surprisingly, primary human macrophages exhibited neither quantitative nor structural changes of the actin and vimentin cytoskeleton after 11 days in microgravity when compared to 1g controls. Neither CD18 or CD14 surface expression were altered in microgravity, however ICAM-1 expression was reduced. The analysis of 74 metabolites in the cell culture supernatant by GC–TOF–MS, revealed eight metabolites with significantly different quantities when compared to 1g controls. In particular, the significant increase of free fucose in the cell culture supernatant was associated with a significant decrease of cell surface–bound fucose. The reduced ICAM-1 expression and the loss of cell surface–bound fucose may contribute to functional impairments, e.g. the activation of T cells, migration and activation of the innate immune response. We assume that the surprisingly small and non-significant cytoskeletal alterations represent a stable “steady state” after adaptive processes are initiated in the new microgravity environment. Due to the utmost importance of the human macrophage system for the elimination of pathogens and the clearance of apoptotic cells, its apparent robustness to a low gravity environment is crucial for human health and performance during long-term space missions.

## Introduction

The hostile environment of microgravity during human spaceflight bears a multitude of limiting factors for human health and performance [1]. In particular, serious concerns whether spaceflight-associated immune system weakening ultimately precludes the expansion of human presence beyond Earth's orbit, arose [2]. Substantial research activities are required to achieve an appropriate integrated risk assessment and management [3]. Therefore, it is crucial to understand the biology of immune modulation under spaceflight conditions and if and how homeostasis of the immune system's cellular machinery is maintained under such circumstances [4]. Knowing the cellular and molecular mechanisms through which gravity influences cell function, is an important prerequisite to understand immune dysfunction at an integrated level. Since the first pioneering *in vitro* studies [5–8], different studies in real and simulated microgravity have confirmed that molecular mechanisms and signal transduction processes are altered in cells of the immune system, including the monocyte/macrophage system (MMS) [9,10]. The MMS is responsible for the first line of innate immune defense against invading pathogens. It represents an effector system for attacking and killing bacteria after activation by T lymphocytes.

In microgravity, cells of the MMS have demonstrated disturbed cytokine release [11,12,13], reduced oxidative burst [14,15], alteration of the cytoskeleton [16] and changes in gene expression associated with their differentiation [17]. During an experiment on the International Space Station (ISS), a reduction of the locomotion ability was observed [18]. During the SIMBOX (Science in Microgravity Box) mission on Shenzhou-8, launched on board a Long March 2F (CZ-2F) rocket from the Jiuquan Satellite Launch Center (JSLC), we recently found a severely disturbed actin cytoskeleton, disorganized tubulin and distinctly reduced expression of CD18, CD36 and MHC-II in macrophageal differentiated U937 cells after 5 days in microgravity [19]. However, due to the limitations of the model system and because of several technical and biological limitations, the results of the SIMBOX mission should be interpreted with caution [19].

It is decisive to know, if all the observed and often very severe changes in cell culture experiments represent–in the worst case–a “cellular disaster” in microgravity or—more favorable—an initiation of a complex adaptational response. Obviously, a large number astronauts has now completed long-term stays in space without suffering from a severe health problem associated to the effects of microgravity [20]. This leads to the hypothesis that a potential disastrous cellular effect is either well compensated by the organism, or prevented at an early stage through cellular adaptation. While the adaptive response of the human physiological systems in microgravity is well described, often adjusting to a new steady state after hours until weeks [20], our knowledge about cellular adaptation is very limited. Although many studies have reported that diverse key cellular functions are altered in microgravity, including cell morphology, proliferation, growth, differentiation, signal transduction and gene expression, the time course of alterations and potential adaptations to the new gravitational environment are widely unknown. One study reported that an initially damaged cytoskeleton reorganized after 20h in simulated microgravity in glia cells [21]. The plethora of *in vitro* experiments investigated changes in the time frame of minutes, to hours, or even days. Importantly, most of these studies were conducted with tumor cells, obviously different from healthy body cells. They are altered in terms of most of the so far in microgravity investigated parameters, particularly in potential gravity-sensitive structures such as the cytoskeleton [22,23].

Isolated cells are an ideal study object to investigate direct microgravity-induced changes without any influence of indirect or secondary effects outside the cellular target structure. In order to characterize the long-term alteration and a potential “steady state” of human cells in microgravity, we established an experimental system using primary human M1 macrophages—instead of a macrophage-like tumor cell line—and exposed them as long as possible to a microgravity environment. As model system, we selected human primary cells with a naturally long lifespan: The *in vivo* lifespan of tissue-resident macrophages is several weeks up to years [24,25], although considerably shorter in *in vitro* culture. However, the experiment time in space was limited by technical, biological and operational constraints and designed according to the margins validated during the preparatory pre-flight test program. Therefore, we established a completely new biological mission scenario for working with primary human macrophages on board of the International Space Station (ISS) in the “Human Macrophages in Microgravity Environment” (CELLBOX-PRIME) experiment, conducted in the U.S. National Lab of the ISS using the “NanoRacks Astrium Centrifuge”(OpNom: NanoRacks BioRack Centrifuge) in Biorack type I standard CELLBOX EUE type IV container from Airbus Defence and Space. Upload and download of the experiment were conducted with SpaceX CRS-3. After fixation in space and landing, we investigated the cytoskeletal architecture, surface molecules for macrophage activation and cell-cell-communication, as well as the metabolite spectrum in the cell culture supernatant. The experiment time was originally planned for three days, but accidentally prolonged to 11d due a technical malfunction, which allowed to study the cellular status beyond the so far established limits.

## Material and methods

### Primary human macrophages

Primary human M1 macrophages were obtained from PromoCell (Heidelberg, Germany) in M1-Macrophage Generation Medium DXF supplemented with HEPES at a final concentration of 25 mM. Cell culture plates were hermetically sealed to avoid spillage and contamination during transport. Complete cell culture sets of primary human M1 macrophages were transported in actively tempered (37°C) closed sterile containers in cabin and connected to internal aircraft power systems on board of Lufthansa flights LH464 from Frankfurt to Orlando on March 11^th^, 2014 and on April 7^th^, 2014.

### Experiment concept

**Fig 1:**
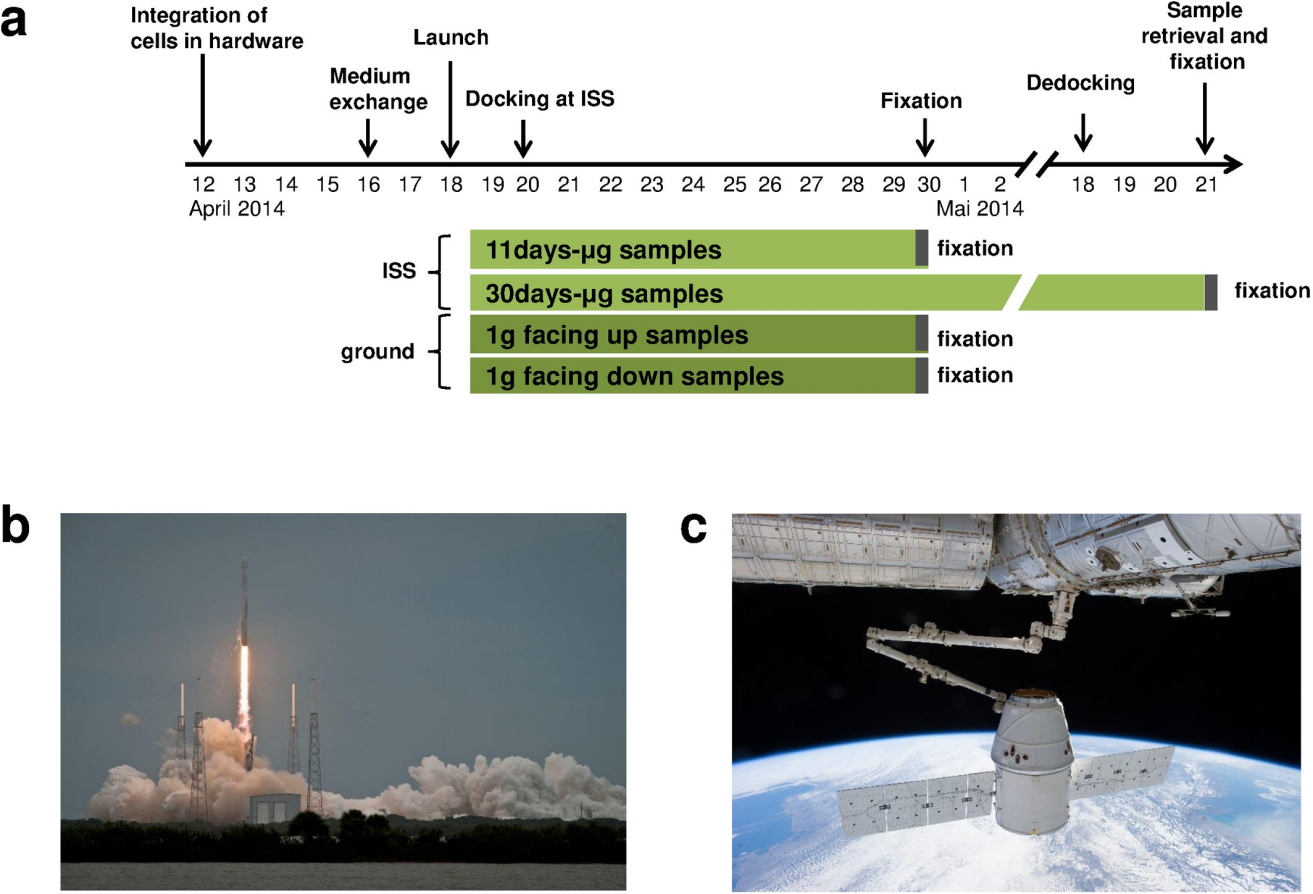
Mission profile of CRS-3 and experimental concept. (a) Mission timeline of the SpaceX CRS-3 mission and experimental concept. The experiment comprised two sample groups which were transported to the ISS. These were exposed to microgravity. Cells of first group were fixed after 11 days of microgravity. The cells of the second group were fixed after retrieval of the cells after 30 days of microgravity. Additionally, two groups were incubated at 1g on ground, one group with cells “facing up” and one group with cells “facing down". (b) Launch of the SpaceX CRS-3 mission on April 18th, 2014, Falcon 9 rocket with Dragon spaceship from Cape Canaveral SLC-40. c. The Dragon spaceship before berthing on April 20th, 2014.

The experiment was designed to investigate the influence of gravity on the cytoskeleton, cell surface molecules and metabolism in primary human macrophages that have been exposed to microgravity for several days compared to those that were cultured under Earth gravity (1g) in similar culture and hardware conditions. Cells were uploaded to the ISS in the Biorack type I standard CELLBOX EUE type IV container (Airbus Defence and Space) and retrieved during the SpaceX CRS-3 mission. On board the ISS, the experiment was integrated into the NanoRacks Laboratory and the “NanoRacks Astrium Centrifuge”(OpNom: NanoRacks BioRack Centrifuge) in the U.S. National Lab. Key steps of the pre-launch phase were 1.) differentiation of primary human macrophages from human peripheral blood monocytic cells on polycarbonate slides in the home laboratories, 2.) transport of materials and liquids for preparation of the hardware to the launch site laboratory nine days before launch, 3.) reserve transport of differentiated primary human macrophages to the launch site laboratory (special in-cabin transport with LH464 from Frankfurt to Orlando) three days before launch, 4.) medium exchange and cell culture in CO2-incubator until one day before launch to the launch site and 5.) integration of cells, fixation and stabilization solution into the flight and ground reference experiment hardware one day before launch. During the ISS mission, addition of fixation solution was planned 72 hours after experiment activation, latest 120 hours after launch, whereas addition of stabilization solution was planned 2h after fixation. Ground reference experiments were performed in parallel at the launch site laboratory (Space Life Sciences Lab, KSC). During the experiment mission, a hardware failure caused a significant alteration of the experiment process, which also influenced the experiment design. Due to a technical failure of hardware control systems on board the ISS, no automatic activation of the experiment was possible after docking (April 20^th^, 2014) and unloading. Initiation of troubleshooting and the search for alternative procedures resulted in a delay of the experiment activation and the centrifuge was not activated. Finally, on April 30^th^ 2014, the experiment containers were inserted into the Biorack Frame and manual activation using MIMO Interface was conducted. Therefore, instead of six experiment unique equipment units (EUEs) fixed after 3d with two in-flight groups of 3 x 0g and 3 x 1g, five EUEs fixed after 11d and one EUE fixed after 30d were returned to Earth. Consequently, the ground reference experiment units (1g ground) were treated and fixed according to the off-nominal in-flight procedures (Fig 1A). To investigate the influence of the gravitational force direction under 1g conditions, cells were cultivated in two different orientations. In the “facing upwards” group, the vector of gravitational force was oriented towards the basal surface where the primary human macrophages were attached to the polycarbonate slide. In the “facing downwards” group, the vector of gravitational force was oriented towards the apical cell surface. In both groups the vector of gravitational force was oriented perpendicular to the slide surface. As classification into "up" and "down" is not applicable in weightlessness, it was not mentioned for the experiment groups on board the ISS. After retrieval of the samples, immunocytochemical staining of surface molecules and cytoskeletal components was performed. Additionally, the metabolite spectrum of the cell culture supernatants was analyzed by GC–TOF–MS.

### Mission profile of SpaceX CRS-3

The experiment was brought to the International Space Station (ISS) on board the third SpaceX cargo resupply mission (CRS-3) contracted by NASA. The experiment was completely prepared four times due to three short-term launch delays and one launch scrub in March and April 2014 and finally prepared for the launch on April 18th, 2014, with a Falcon 9 type rocket with the Dragon spacecraft from Cape Canaveral SLC-40, Florida (Fig 1B). Docking to the ISS was executed on April 20^th^ (Fig 1C), and the spacecraft stayed berthed to the ISS until May 18th, 2014. After deorbiting, the spacecraft came down in the Pacific Ocean offshore on the coast of California, USA on the same day.

### Experiment hardware and implementation on the ISS

**Fig 2:**
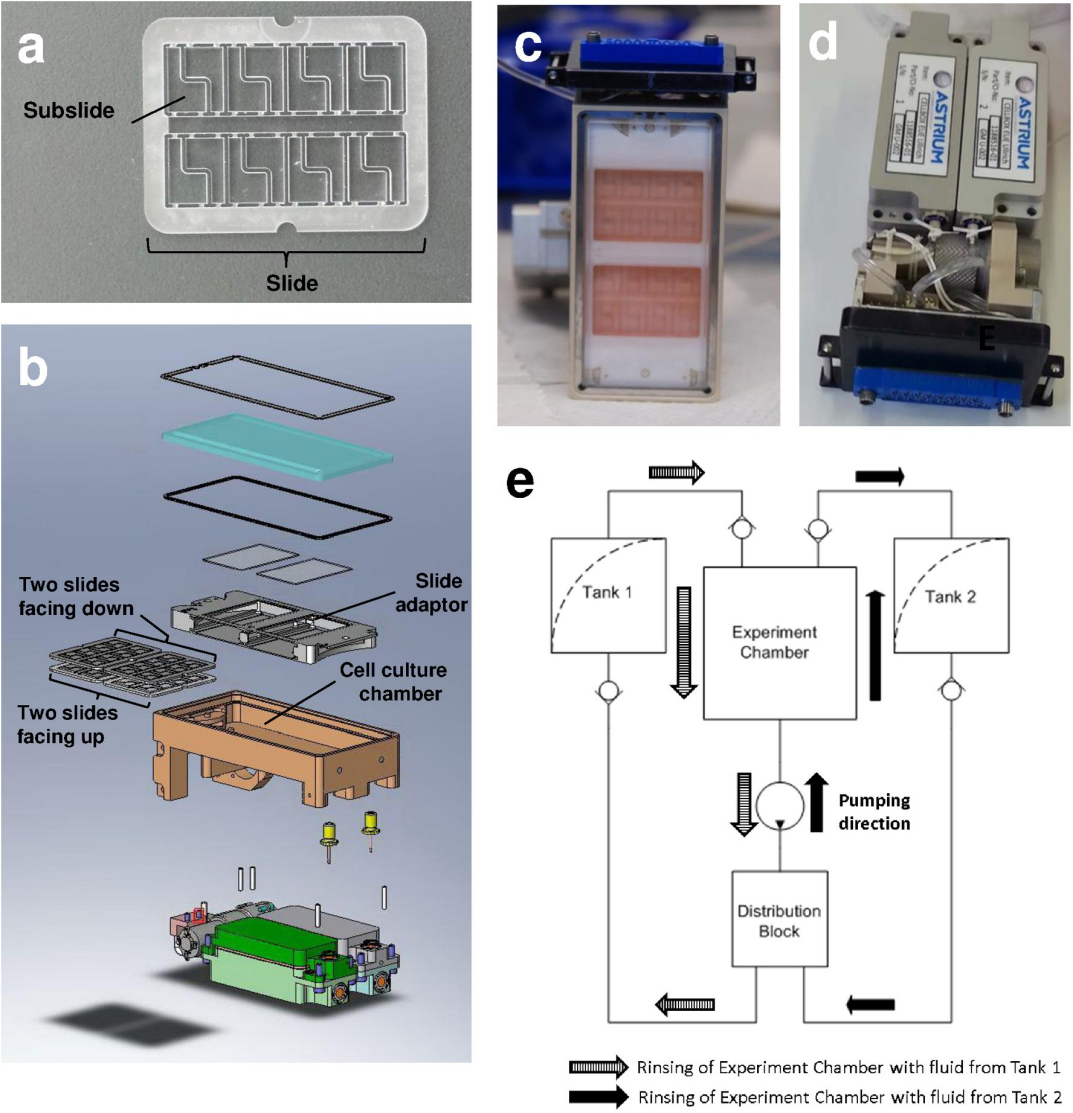
In-flight hardware: Biorack type I standard CELLBOX EUE type IV container. (a) Polycarbonate slide serving as cultivation surface for primary human macrophages. The slides are segmented into 16 subslides. (b) Individual parts of the experiment core. (c) Assembled experiment core. View through the transparent lid on the cell culture chamber with the polycarbonate slides. (d) Assembled experiment insert, view on tanks. (e) Scheme of fluid exchanges. Pump can operate in both directions; check-valves determine from which of the two tanks liquid is transported into the cell culture chamber.

**Fig 3:**
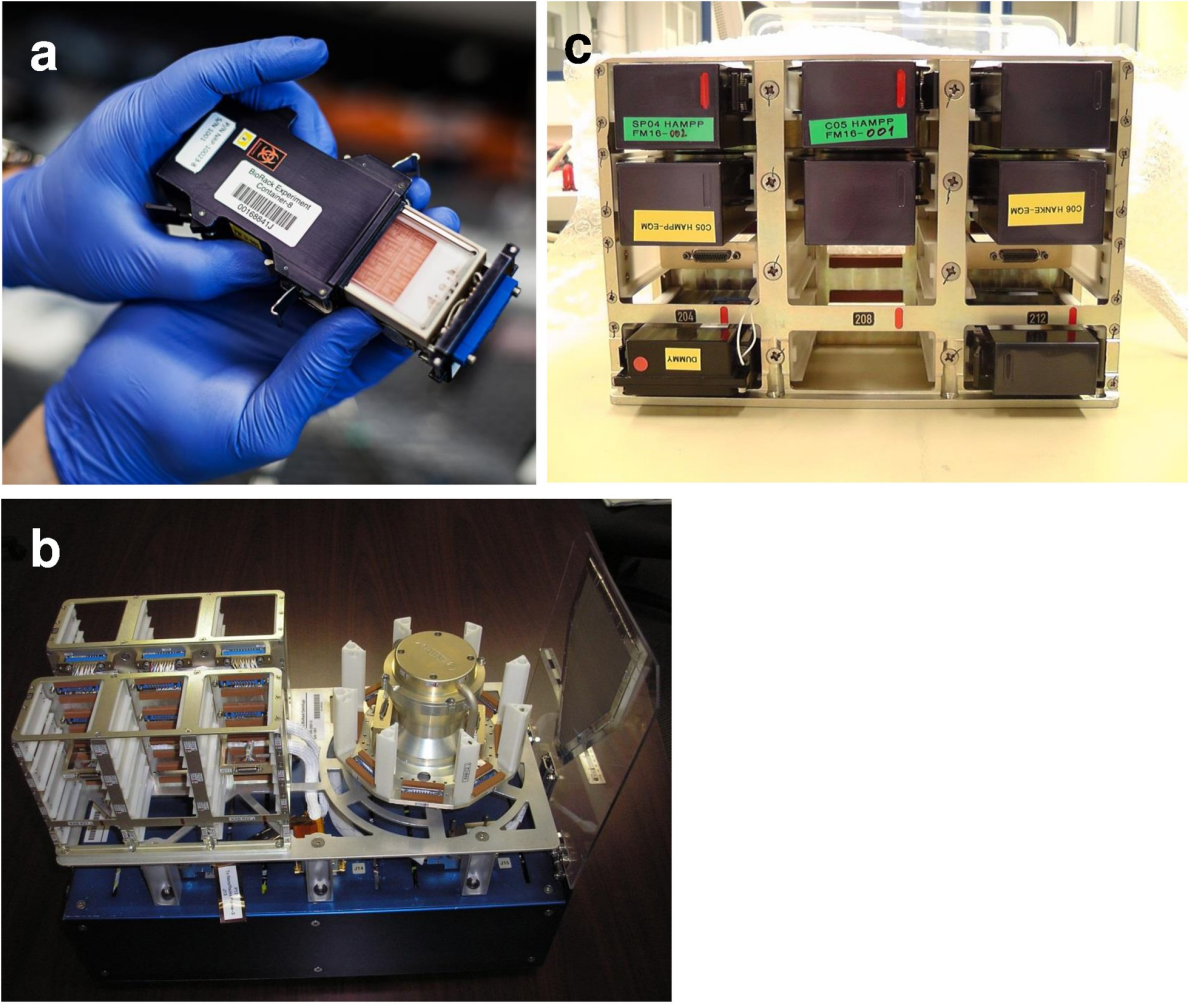
“NanoRacks Astrium Centrifuge” (U.S. National Laboratory) containing Biorack type I standard CELLBOX EUE type IV (Photos: Jesper Rais). (a) Biorack type I container with inserted CELLBOX EUE type IV. (b) “NanoRacks Astrium Centrifuge” with static and centrifuge slots. (c) “NanoRacks Astrium Centrifuge” with integrated EUE inserts.

“Biorack Experiment Inserts Typ IV” developed by Airbus DS (Friedrichshafen, Germany) were used. These experimental units enable the cultivation of adherent cells on polycarbonate slides in a medium-containing and temperature controlled cell culture chamber (Fig 2). The slides were segmented into 16 L-shaped sections that could be separated after the experiment, allowing for 16 independent analyses from one slide. Slides with adherent cells were implemented in the hardware and held in place by a slide adapter. The slide adapter holds four slides in each unit (Fig 2B and 2C). Cells were oriented towards the center of the cell culture chamber to ensure optimal medium supply, two slides “facing up” and two slides “facing down” (Fig 2B). The experiment inserts enabled two fluid exchanges in the cell culture chamber at arbitrary time points. For this purpose the culture chambers were connected to two tanks (Fig 2B and 2D). Each tank had two compartments that were separated by a flexible partition. An integrated pump transported the liquid from one tank-compartment into the cell culture chamber, while the previous fluid from the cell culture chamber was displaced into the other tank-compartment (Fig 2E). On the ISS, the units were implemented in the NanoRacks Lab of the U.S. National Lab of the ISS (Fig 3A–3C). The unit comprised stationary slots for exposure to microgravity and slots on a centrifuge to provide gravitation on the ISS (Fig 3B and 3C).

### Experiment preparation and protocols

The primary human macrophages were transported from Heidelberg, Germany, to the Space Life Science Labs (Exploration Park, Kennedy Space Center, Florida) on board of Lufthansa flight LH464 in-cabin. The transport cell medium was supplemented with 25 mM HEPES for additional buffering without CO_2_ administration. Transport-boxes (modified Waeco TC-35) connected to the aircraft internal power systems provided a constant temperature of 37°C throughout the entire transport. Additional external batteries ensured continuous power supply.

All components of the hardware were sterilized by ethylene oxide exposure or autoclaving. Experiment units were assembled, and the primary human macrophages on polycarbonate slides were implemented six days before launch under sterile conditions. Six experimental units (each holding four slides) were prepared for the flight to the ISS, five units were fixed after 11 days of microgravity, and one unit was fixed after retrieval (Fig 1A). Additionally, two units were assembled for ground 1g reference control experiments with cells from the same donor as the flight samples. Four days before launch, the medium in the cultivation chamber of all units was exchanged to supply the cells with fresh medium before the launch (Fig 1A). Between assembly and loading into the Dragon spaceship, the experimental units were stored at 37°C. From then on until unloading the units on the ISS, the temperature was kept passively at ambient, but not below 20°C or higher than 37°C. After 11 days of microgravity, the cells of the respective experiment groups (11 days microgravity and both 1g control groups) were fixed by pumping PBS with 2% paraformaldehyde and 1.3% sucrose from the first tank of the hardware into the cell culture chamber. 120 minutes later, PBS was pumped into the cell culture chamber to remove the paraformaldehyde solution and thus to avoid over-fixation. After fixation, experiment units were removed from the Biorack frame and stored at 4°C until download. After the landing of the Dragon spaceship in the Pacific Ocean, the experimental units were unloaded and transported to the Space Life Sciences Labs (Exploration Park, KSC, Florida). The 30 days microgravity samples were fixed using the same fixation regime that was used for the other sample groups. The hardware was disassembled, cells on slides were stored in PBS and cell culture supernatant for analysis of metabolites was recovered from the respective compartment of the hardware tanks. All samples (flight, ground and cell culture control samples) were transported on board of Lufthansa flight LH465 from Orlando to Frankfurt and by car to Zurich, in an actively cooled transport box (modified Waeco TC-35) at 4°C until analysis.

### Immunocytochemical staining

The polycarbonate slides were separated into 16 separate subslides (Fig 2A) which were used for the immunocytochemical stainings. In addition to the epitope of interest, the samples were co-stained for apoptosis with TUNEL™ (APO-BRDU™ Kit, BD Biosciences, Allschwill, Switzerland), for cytoplasm with CellMask (HCS CellMask™ Blue Stain, Molecular Probes), and for the nucleus with DRAQ5 (CellSignalling). The cells were permeabilized by 15 minutes incubation in 0.1% saponin (Sigma Aldrich, Buchs, Switzerland) in PBS (Biochrom, Berlin, Germany) followed by three 5 minutes washes in PBS. Subsequently, the samples were incubated for three hours in TdT Enzyme/Br-dUTP labeling solution (TUNEL). Then, the samples were blocked using 1% BSA (Fluka) in PBS and incubated with primary antibodies or phalloidin. Blocking time, antibody-incubation time and dilution were: anti-CD18, (Acris, Herford, Germany) dilution 1:50, incubation overnight, blocking 1 hour; anti-ICAM, (BD Biosciences), dilution 1:100, incubation overnight, blocking 1 hour; anti-CD14, (Biolegend, San Diego, USA), dilution 1:50, incubation 2 hours, blocking 30 minutes; actin stain (phalloidin) (Invitrogen), dilution 1:40, incubation 1 hour, blocking 1 hour; anti-vimentin (Abcam, Cambridge, UK), dilution 1:40, incubation overnight, blocking 2 hours. The samples were washed three times for 5 minutes in PBS. Subsequently, the samples were incubated for 1 hour in secondary antibody goat anti-mouse or goat anti-rabbit Alexa 568 labeled (Invitrogen), dilution 1:1000, and anti-BrdUTP FITC labeled (eBioscience, San Diego, USA) (to label TUNEL reaction), dilution 1:40 in 0.5% BSA in PBS. The samples were washed three times for 5 minutes in PBS. Then, they were incubated in DRAQ5, 1:500 in 0.5% BSA in PBS for 30 minutes followed by three washing steps as above. Thereafter, cells were stained with CellMask, 1:2500 in PBS for 30 minutes. After further three washing steps as described above, the subslides were fixed upside down onto glass slides with Immunomount (DAKO), dried for 12–24 hours and subjected to microscopy.

### Staining of surface-bound fucose

The polycarbonate sections were removed from whole slides and stained for surface-bound fucose using a lectin-biotin-streptavidin system. In brief, the sections were washed 3 times for 5 minutes with PBS and then blocked with 1% BSA for 30 minutes at room temperature. The sections were washed again 3 times with PBS and incubated with 6.4 μg/ml biotinylated Aleuria Aurantia Lectin (AAL) (VECTOR Laboratories) for 30 minutes at room temperature. After 3 times washing with PBS, the sections were incubated with 10 μg/ml fluorescently conjugated streptavidin (Streptavidin, Alexa Fluor 568 conjugate, life technologies) for another 30 minutes at room temperature. After washing with PBS again, the sections were embedded on coverslips in mounting medium (Immunomount, DAKO).

### Confocal microscopy

The labeled cells were imaged using a Leica SP5 confocal microscope and Leica software „Application Suite X Advanced Fluorescence”(Leica Microsystems, Wetzlar, Germany). Six to eight images, randomly distributed over the whole slide, were taken from each subslide. The resolution was chosen as 12 bits, the format width and height were 512x512 pixels. Scan speed was 700 Hz. A 40x/1.25 oil lens and HyD detector were used. Alexa Fluor 568 signals were detected with a laser at 561 nm (emission at 573–639 nm), nuclear staining (DRAQ5) was detected with a laser at 633 nm (emission at 653–714 nm), plasma membrane staining (HCS CellMask Blue) was detected with a laser at 405 nm (emission at 417–511 nm), and TUNEL staining for apoptosis screening was detected with a laser 488 nm (emission at 493–586 nm).

### 3D analysis of structure filaments

Vimentin and actin were stained by immunofluorescence as described above. The cells were imaged by confocal laser scanning microscopy (Leica SP5). The cells were analyzed in X/Y/Z stacks. Stack distance was at most 0.2 μm. 3D pictures were merged from up to one hundred single planes for the 3D analysis by Imaris software (Bitplane). Three independent persons evaluated the reorganized 3D pictures optically and graded each cell according to the following criteria: a) staining of single cells (homogenous/non-homogenous/unstained), b) cells size, and c) filament structure (strings, cluster big/small, clouds, unstained cells). Only cells positive for HCS CellMask Blue and negative for TUNEL staining were analyzed. These represent the living cell population. Between 50 cells (30d-μg group) and 250 cells (11d-μg group, 1g control group, cell culture control) were analyzed per group.

### Widefield microscopy

Labeled cells were imaged using a DMI 6000 Leica fluorescent microscope and Leica software „Application Suite X Advanced Fluorescence”(Leica Microsystems, Wetzlar, Germany). Eight images in consecutive order, starting at the border of the subslide going towards the center, were taken from each subslide. Alexa Fluor 568 signals were detected with the Leica RFP fluorescent filter (excitation at 546 nm, emission at 605 nm), nuclear staining (DRAQ5) was detected with the CY5 filter (excitation at 620 nm, emission at 700 nm) and plasma membrane staining (HCS CellMask Blue) was detected with the A4 filter (excitation at 360 nm, emission at 470 nm). All images of one specific epitope were taken with identical settings for intensity, exposition and gain.

### Quantitative analysis of microscopic images

Images recorded by widefield or confocal microscopy (LIF Files) were analyzed and measured with the image processing software Imaris 7.7.1 (Bitplane). At least three subslides from each experiment group were analyzed. Eight images were taken from each subslide comprising about 15 cells per image, resulting to about 360 analyzed cells per experimental group. Measured parameters were the cell area, the cell number per picture and the relative fluorescent intensity (RFI) of the particular epitope of each stained cell. The area of the cells and the cell number per area were both estimated with the use of the HCS CellMask Blue staining. A template for the cell area was defined, and seed points were used to identify cells. The threshold for absolute intensity was set. Filters used for this determination were “Quality above threshold” and “Distance to border”. Unstained cells or false positive and unspecific signals were excluded manually. After determination of the cell area of each cell, the RFI of the epitope staining was calculated using the HCS CellMask Blue-deduced area and the signal from the respective epitope staining. Background signal was measured and subtracted. Parameters had to be adapted for every single image analyzed.

### Metabolite analysis

Metabolites for GC–TOF–MS were extracted and derivatized using modified methods [26–28]. For the extraction 100 μl of supernatant was mixed with 900 μl cold (-20°C) 80% methanol containing 20 μl ribitol (0.2 mg ml-1 in water) and 10 μl 13C-sorbitol (0.2 mg ml-1 in water), which were added as internal standards for the quantification of metabolite abundances. After incubation at 21°C for 10 min, the extract was centrifuged for 15 min at 25000 g. For further analysis, 50 μl of the supernatant was dried in vacuo. The pellet was resuspend in 10 μl of methoxyaminhydrochloride (20 mg ml-1 in pyridine) and derivatized for 90 min at 37°C. After the addition of 20 μl of BSTFA (N,O-Bis[trimethylsilyl]trifluoroacetamide) containing 5 μl retention time standard mixture of linear alkanes (n-decane, n-dodecane, n-pentadecane, n-nonadecane, n-docosane, n-octacosane, n-dotriacontane), the mix was incubated at 37°C for further 45 min. A volume of 1 μl of each sample was injected into a GC–TOF–MS system (Pegasus HT, Leco, St Joseph, USA). Samples were derivatized and injected by an autosampler system (Combi PAL, CTC Analytics AG, Zwingen, Switzerland). Helium acted as carrier gas at a constant flow rate of 1 ml/min. Gas chromatography was performed on an Agilent GC (7890A, Agilent, Santa Clara, USA) using a 30 m VF-5ms column with 10 m EZ-Guard column. The injection temperature of the CIS injector (CIS4, Gerstel, Mühlheim, Germany) increased with a rate of 12°C s-1 from initially 70°C to finally 275°C. Transfer line and ion source were set to 250°C. The initial oven temperature (70°C) was permanently increased to a final temperature of 320°C by 9°C per minute. To avoid solvent contaminations the solvent delay was set to 340 s. Because of the chemical and physical properties of the different metabolites the mixture was separated on the column over time. Metabolites that passed the column were released into the TOF-MS. The transferline, connecting the GC and the TOF-MS, was set to 250°C as well as the ion source where the in streaming metabolites got ionized and fractionated by a pulse of 70 eV. Charged mass fragments were flown through the vacuum flight tube until they reached the mass detector. Each fragment had a specific time of flight, depending on its mass charge ratio (m/z) until its impact on the detector. Mass spectra were recorded at 20 scans per second with an m/z 35–800 scanning range. Chromatograms and mass spectra were evaluated using the ChromaTOF 4.5 and the TagFinder 4.1 software [29].

### Statistical analysis

*Immunocytochemistry*: Statistical evaluation of two groups was performed using the Mann Whitney test. Three or more groups were compared by Kruskal-Wallis and Dunn’s Multiple comparison test. The software GraphPad Prism 5.0 (GraphPad software Inc., San Diego, CA) was used. *Metabolites*: Each relative metabolite abundance (RMA) was multiplied by the recovered volume of the respective experiment compartment. These "absolute" RMAs of one experiment unit were summed up to yield the total value of the metabolite within one entire experimental unit. Then, the mean for each metabolite was calculated out of all flight modules and ground modules, respectively. Using GraphPad Prism software a nonparametric Mann-Whitney test was performed comparing the mean values of each metabolite of the flight modules to the mean values of the ground modules to identify significant differences between supernatants fixed on orbit and on ground.

## Results

### Increased cell area and cell number after 11 days of microgravity

**Fig 4:**
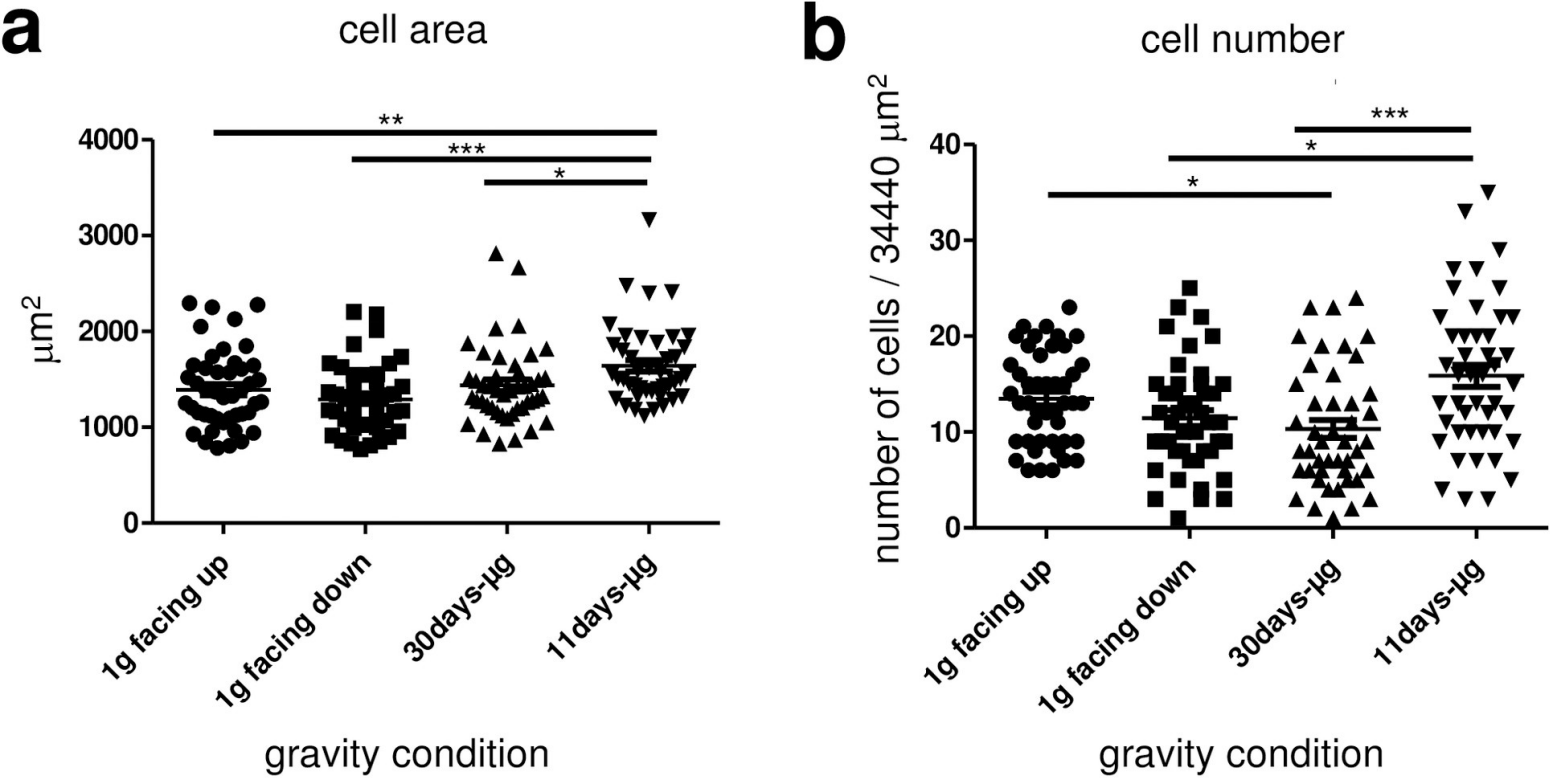
Quantification of cell area and cell number of primary human macrophages exposed to different gravity conditions. (a) The cell area was measured by immunofluorescent staining of the cell membrane and subsequent half-automated assessment of the stained area. Data points represent the mean area of all cells on one picture. 45 microscopic pictures (464–607 cells) were analyzed per gravity condition. Sample groups 1g facing down (1293 +/- 52.48 μm2, n = 45), 1g facing up (1391 +/- 59.93 μm2, n = 45), 30d-μg (1437 +/- 60.04 μm2, n = 45) and 11d-μg (1643 +/- 59.6 μm2, n = 45) are demonstrated. Single data points and means are shown for each experimental group (*p<0.1, **p<0.05, ***p<0.005). (b) The cell number was measured by fluorescent staining of the cell membrane and subsequent half-automated cell-counting. Data points represent the number of all cells on one picture. 45 microscopic pictures were analyzed per gravity condition. Sample groups 1g facing down (11.44 +/- 0.84, n = 45), 1g facing up (13.49 +/- 0.721, n = 45), 30d-μg (10.31 +/- 0.9265, n = 45) and 11d-μg (15.48 +/- 1.184, n = 44) are demonstrated. Single data points and means are shown for each experimental group (*p<0.1, **p<0.05, ***p<0.005).

The density of the cells on the slide surface, and the surface area of the single cells were measured to detect any changes due to gravity conditions. Quantification of the cell area revealed a significant 18% increase in the 11d-μg samples (n = 45) compared to the 1g facing up samples (n = 45) and a significant 7% increase compared to the 1g facing down samples (n = 45) (Fig 4A). The cell area of the 11d-μg samples (n = 45) was 14% larger than the cell area of the 30d-μg samples (n = 45). No other significant differences regarding cell area were found between the groups (Fig 4). Cell number of the 11d-μg samples (n = 44) was significantly increased for 35% when compared to the 1g facing down samples (n = 45) (Fig 4B). The number of the 1g facing up cells were 30% higher than in the 30d-μg group (n = 45) (Fig 4B). Therefore, cell area and number increased after 11days in microgravity.

### Decreased expression of the cell adhesion molecule ICAM-1 and no alterations of CD18 in microgravity

**Fig 5:**
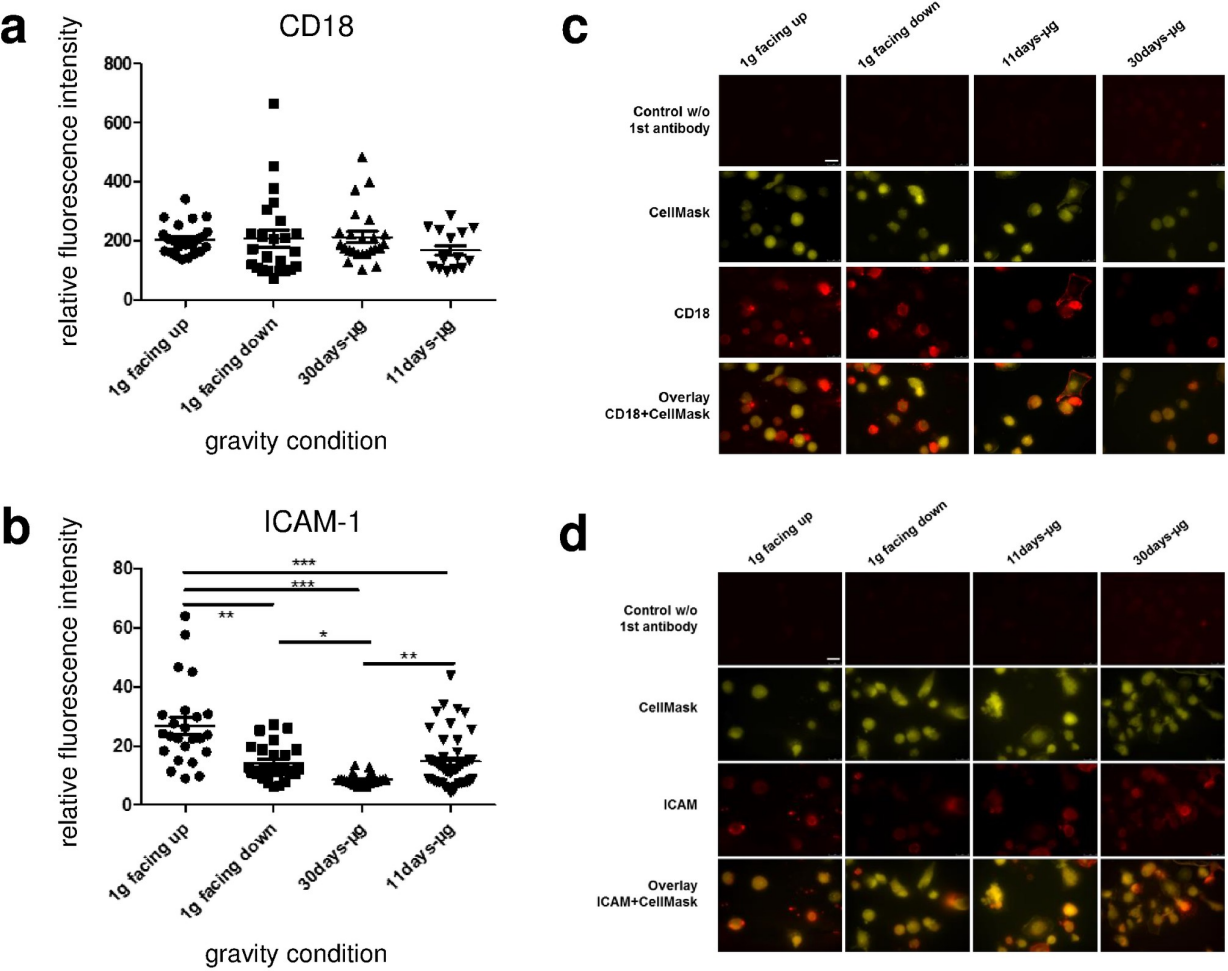
Quantification of surface-bound CD18 and ICAM-1 of primary human macrophages exposed to different gravity conditions. Surface-bound CD18 and ICAM-1 were visualized by immunofluorescent staining and subsequently analyzed quantitatively. Data are expressed as relative fluorescent intensity (RFI) and represent the mean RFI of all cells on one picture. (a) CD18: 16–24 pictures (187–390 cells) were analyzed per gravity condition. (b) ICAM-1: 24–48 pictures (240–838 cells) were analyzed per gravity condition. Sample groups 1g facing up (26.84 +/- 2.87, n = 24), 1g facing down (14.22 +/- 1.276, n = 24), 30d-μg (8.533 +/- 0.3942, n = 24) and 11d-μg (14.67 +/- 1.28, n = 48) are demonstrated. Single data points and means are shown for each experimental group (*p<0.1, **p<0.05, ***p<0.005). (c) Microscopic images of primary human macrophages exposed to different gravity conditions. CD18 was stained (red) and the cytoplasm was stained with CellMask (yellow). Controls without anti-CD18 antibody and an overlay of CD18 and CellMask are shown. Scale-bar = 25 μm. (d) Microscopic images of primary human macrophages exposed to different gravity conditions. ICAM-1 was stained (red) and the cytoplasm was stained with CellMask (yellow). Controls without anti-ICAM antibody and an overlay of ICAM-1 and CellMask are shown. Scale-bar = 25 μm.

To detect possible changes in the ability of cells to adhere to each other upon different gravitational conditions, macrophages were stained for cell adhesion molecules CD18 and ICAM-1 (Fig 5). As shown in Fig 5A, quantification of CD18 staining by relative fluorescent intensity (RFI) revealed no significant differences between the different gravity conditions. The lowest intensities were measured in the 11d-μg group (n = 16). As shown in Fig 5B, analysis of ICAM-1 revealed significantly 45% lower RFI in the 11d-μg samples (n = 48) compared to the 1g facing up samples (n = 24) (Fig 5B). Interestingly, the RFI was also 47% lower in the 1g facing down group (n = 24) compared to the 1g facing up group (n = 24). ICAM-1 staining intensity was 41% lower in the 30d-μg samples (n = 24) compared to the 11d-μg samples (n = 48), as well as 68% lower compared to the 1g facing up samples (n = 24) and 60% lower compared to the 1g facing down samples (n = 24). In the 11d-μg samples (n = 48), ICAM-1 was expressed 45% lower than in the 1g facing up samples (n = 24). Thus, ICAM-1 cell surface expression was dependent on the gravitational force and was reduced in microgravity.

### Surface CD14 expression depends on the direction of the gravity vector

**Fig 6:**
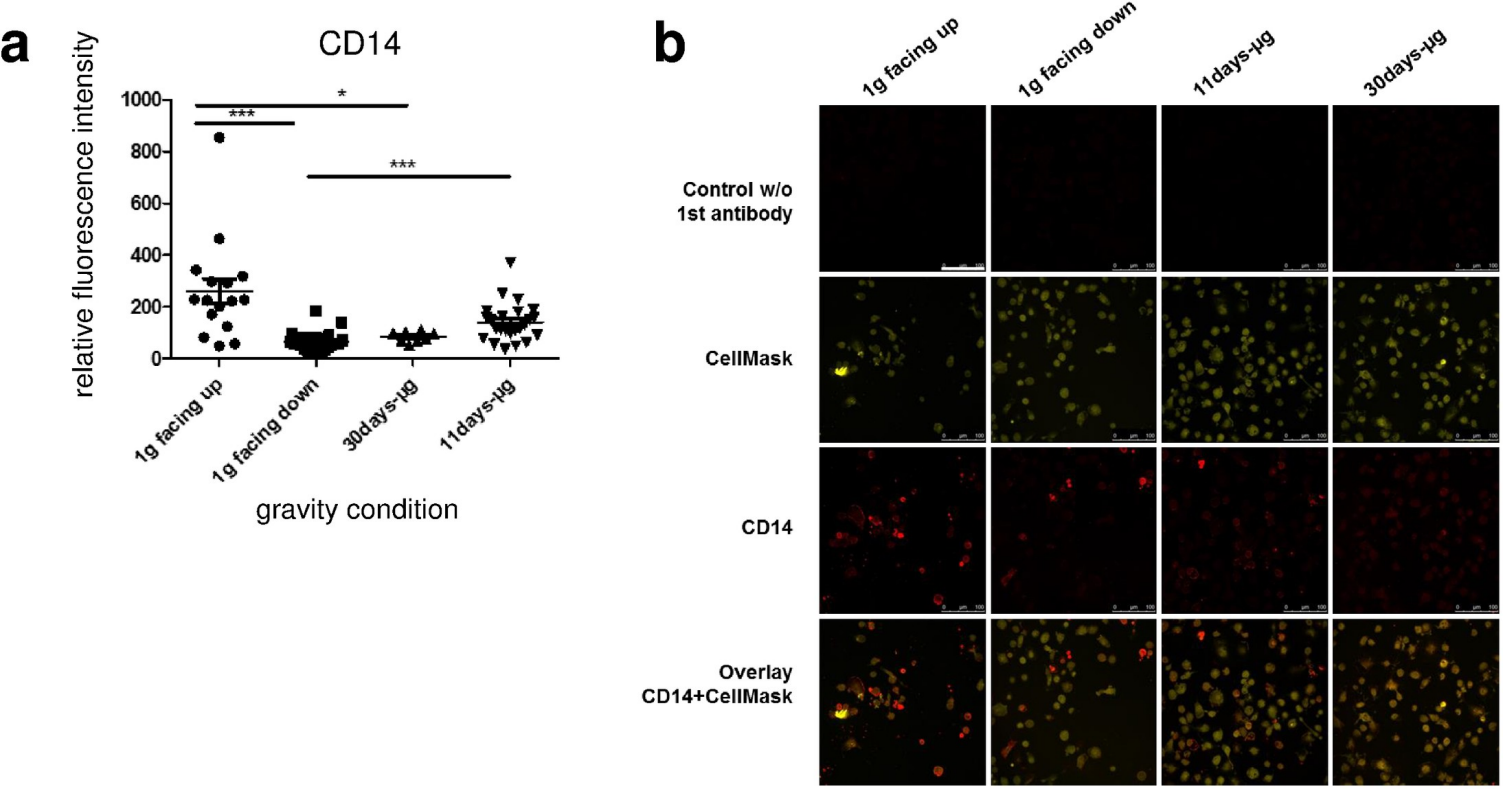
Quantification of surface-bound CD14 of primary human macrophages exposed to different gravity conditions. Surface-bound CD14 was measured by immunofluorescence staining and subsequent quantitative analysis. (a) Data were expressed as relative fluorescent intensity (RFI). Data points represent the mean RFI of all cells on one picture. 8–24 pictures (431–2776 cells) were analyzed per gravity condition. Samples groups 1g facing up (260.8 +/- 48.3, n = 16), 1g facing down (64.48 +/- 7.715, n = 24), 30d-μg (84.54 +/- 7.435, n = 8) and 11d-μg (139 +/- 15.11, n = 24) are demonstrated. Single data points and means are shown for each experimental group (*p<0.1, **p<0.05, ***p<0.005). (b) Microscopic images of primary human macrophages exposed to different gravity conditions. CD14 was stained (red) and the cytoplasm was stained with CellMask (yellow). Controls without anti-CD14 antibody and an overlay of CD14 and CellMask are shown. Scale-bar = 100 μm.

To detect possible changes in the abundance of pattern recognition receptors upon different gravitational conditions, primary human macrophages were stained for CD14 (Fig 6). Comparison of RFI revealed a significant increase of 115% in the 11d-μg samples (n = 24) in contrast to the 1g facing down samples (n = 24). Interestingly, CD14 expression in the 1g facing up group (n = 16) was 304% higher than in the 1g facing down group (n = 24). The 30d-μg samples (n = 8) showed a 67% decreased CD14 expression compared to the 1g facing up samples (n = 16), but were comparable with the 1g facing down group (n = 24) (Fig 6). Therefore, cell surface expression of CD14 was dependent on the direction of the gravitational force.

### No quantitative changes of the actin and vimentin cytoskeleton after 11 days of microgravity

**Fig 7:**
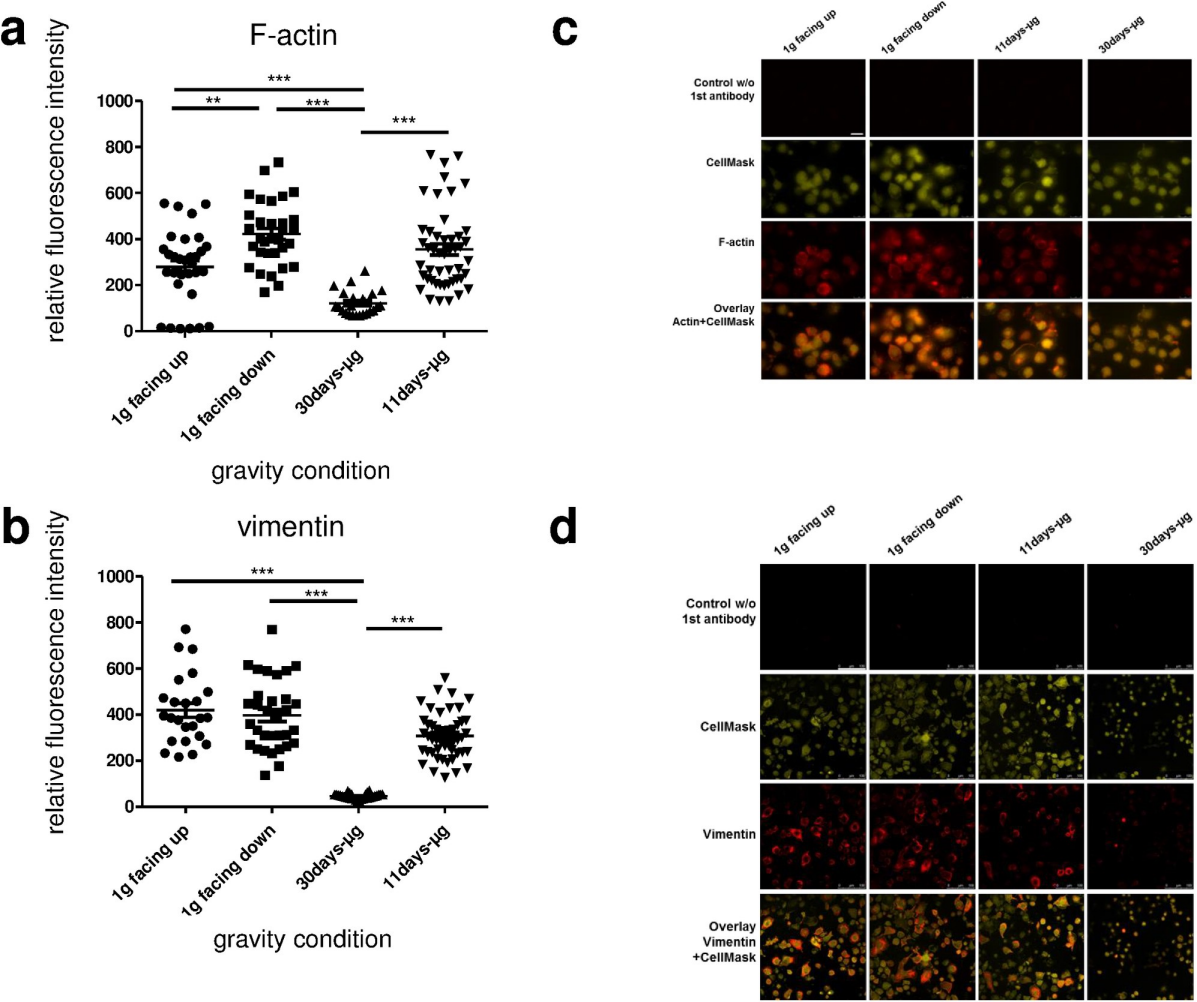
Quantification of actin and vimentin of primary human macrophages exposed to different gravity conditions. Cytoskeletal actin and vimentin was measured by immunofluorescence staining and subsequent quantitative analysis. Data are expressed as relative fluorescent intensity (RFI). Data points represent the mean RFI of all cells on one picture. (a) F-actin: 24–48 pictures (491–1096 cells) were analyzed per gravity condition. Sample groups 1g facing up (278.5 +/- 28.31, n = 32), 1g facing down (421.9 +/- 24.56, n = 32), 30d-μg (121.3 +/- 10.67, n = 24) and 11d-μg (355 +/- 24.73, n = 48) are demonstrated. Single data points and means are shown for each experimental group (*p<0.1, **p<0.05, ***p<0.005). (b) Vimentin: 24–48 pictures (1944–4316 cells) were analyzed per gravity condition. Sample groups 1g facing up (419.2 +/- 30.85, n = 24) 1g facing down (396.5 +/- 26.6, n = 32), 30d-μg (45.88 +/- 2.00, n = 32) and 11d-μg (307.6 +/- 14.14, n = 48) are demonstrated. Single data points and means are shown for each experimental group (*p<0.1, **p<0.05, ***p<0.005). (c) Microscopic images of primary human macrophages exposed to different gravity conditions. F-actin was stained with phalloidin (red) and the cytoplasm was stained with CellMask (yellow). Controls without phalloidin and an overlay of actin and CellMask are shown. Scale-bar = 25μm. (d) Microscopic images of primary human macrophages exposed to different gravity conditions. Vimentin was stained (red) and the cytoplasm was stained with CellMask (yellow). Controls without anti-vimentin antibody and an overlay of vimentin and CellMask are shown. Scale-bar = 100 μm.

Cells were stained for F-actin and vimentin to determine possible quantitative changes of cytoskeletal components under different gravity conditions (Fig 7). The measurement of the F-actin staining revealed no significant difference between the RFI of the 11d-μg samples (n = 48) and the 1g samples, neither compared to 1g facing up (n = 32) nor to 1g facing down group (n = 32) (Fig 7A). Regarding vimentin, no statistically significant or visual difference between 1g facing up (n = 24) and 1g facing down samples (n = 32) could be detected (Fig 7B). The 11d-μg samples (n = 48) appears visually, but not statistically significantly different from the 1g samples (facing up and facing down). The 30d-μg samples showed a significant and striking decrease in actin and vimentin staining intensity compared to all other groups. Therefore, no quantitative changes of the actin and vimentin cytoskeleton could be detected after 11 days of microgravity.

### No significant structural changes in the actin and vimentin cytoskeleton after 11 days of microgravity

**Fig 8:**
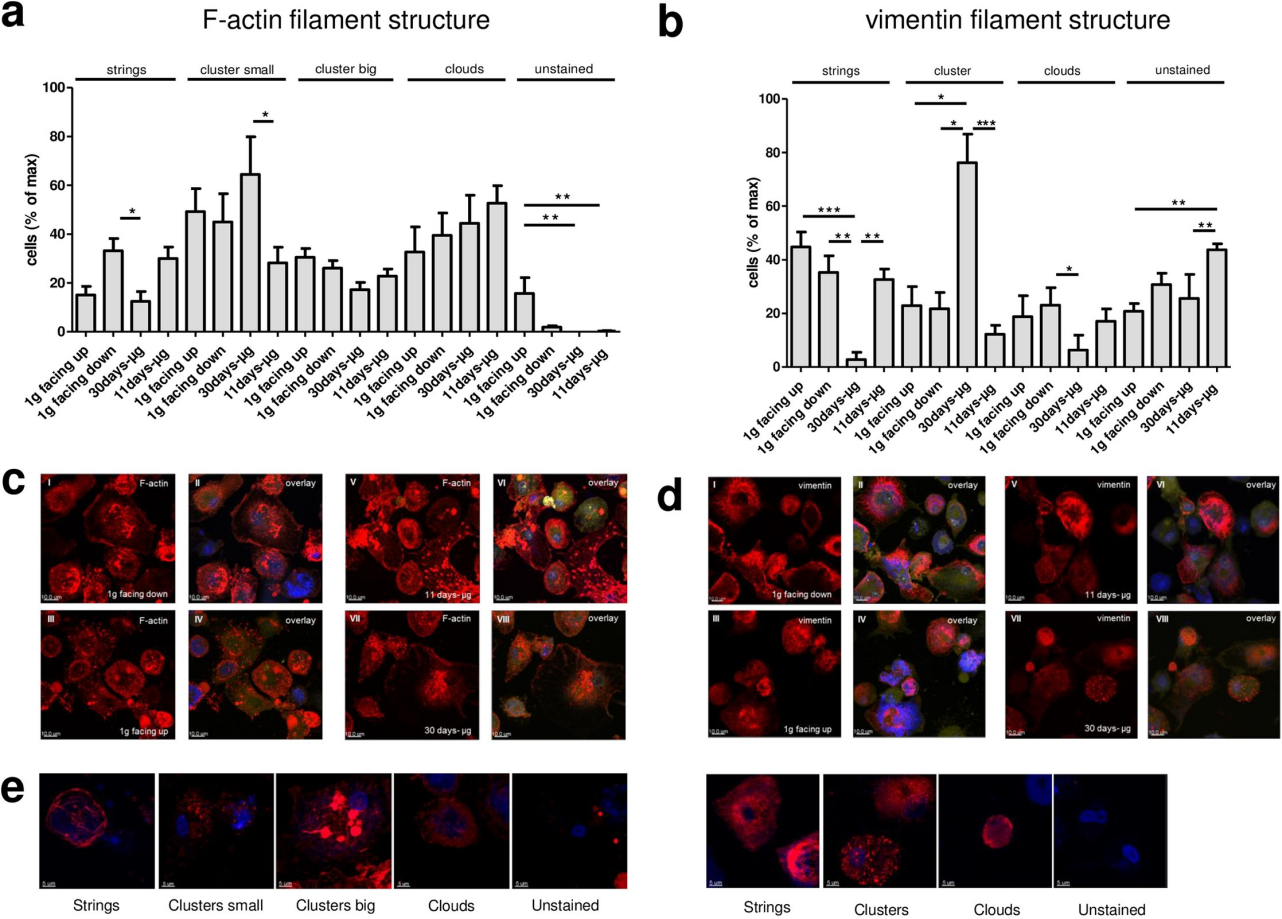
Confocal microscopy analysis of the cytoskeletal structure. (a) Qualitative analysis of cytoskeletal F-actin: 130–146 cells were analyzed per experimental group. Bars represent the percentage of the cells in which the indicated micromorphology of F-actin was visible (strings, small clusters, big clusters, clouds, or no F-actin-staining). The percentage of cells containing filamentous actin is much higher in the 1g-facing down control group than in the 30d-μg group. There is a smaller number of cells with actin clusters in the 11d-μg group compared to the 30d-μg group. Means and standard errors are shown for each experimental group (*p<0.1, **p<0.05, ***p<0.01). (b) Qualitative analysis of cytoskeletal vimentin: 46–245 cells were analyzed per experimental group. Bars represent the percentage of the cells in which the indicated micromorphology of F-actin was visible (strings, clusters, clusters, clouds, or no vimentin-staining). Means and standard errors are shown for each experimental group (*p<0.1, **p<0.05, ***p<0.01). (c) Representative pictures of cytoskeletal actin staining: The experimental groups "1g-facing down" (I-II), "1g-facing up" (III-IV), "11 days-μg" (V-VI), and "30 days-μg" (VII-VIII) are shown. Only HCS CellMask Blue-positive and TUNEL staining-negative cells were analyzed. Single stain of actin (I, III, V, VII) and overlay of all stainings (II, IV, VI, VIII) (green: TUNEL staining, yellow: HCS CellMask, blue: DRAQ5, red: filamentous actin staining with phalloidin-Alexa Fluor 568). (d) Representative pictures of cytoskeletal vimentin staining: Confocal microscopy analysis of the cytoskeletal protein vimentin. The experimental groups "1g-facing down" (I-II), "1g-facing up" (III-IV), "11 days-μg" (V-VI), and "30 days-μg" (VII-VIII) are shown. Only HCS CellMask Blue-positive and TUNEL staining-negative cells were analyzed. Single stain of cytoskeletal vimentin (I, III, V, VII) and overlay of all stainings (II, IV, VI, VIII) (green: TUNEL staining, yellow: HCS CellMask Blue, blue: DRAQ5, red: cytoskeletal vimentin stained with mouse anti-vimentin and anti-mouse Alexa Fluor 568). (e) Representative pictures of the different micromorphological appearances of the vimentin an actin which were used for the quantification shown in fig 8a and b. (blue: DRAQ5, red: cytoskeletal vimentin stained with mouse anti-vimentin and anti-mouse Alexa Fluor 568 / filamentous actin stained with phalloidin-Alexa Fluor 568).

As shown in Fig 8A and 8B, no significant structural changes in the actin and vimentin cytoskeleton could be observed after 11 days in microgravity. However, after 30 days in microgravity, F-actin strings in the 30d-μg group (n = 9) were 62% reduced compared to the 1g facing down group (n = 12). The number of small clusters was 56% reduced in the 11d-μg group (n = 18) compared to the 30d-μg group (n = 9). As shown in Fig 8C and 8D, the structure of vimentin changed significantly after 30 days in microgravity, whereas no significant changes could be observed after 11 days in microgravity. The percentage of cells with vimentin-strings was very low in 30d-μg group (n = 12) compared to the 1g-facing up (n = 9) or 1g-facing group (n = 12). Vimentin filaments were 92% reduced in the 30d-μg group (n = 12) compared to the 11d-μg group (n = 12), whereas clusters were 3.3-fold higher in the 30d-μg group (n = 12) compared to the 1g-facing up group (n = 9) and 3.5-fold higher in the 1g-facing down group (n = 12). After 30 days of μg (n = 12), the quantity of cells with vimentin clouds was 37% reduced compared to 11 days of μg (n = 18). The latter was comparable to the 1g-facing down group (n = 12) and the 1g-facing up group (n = 9). Whereas after 30d microgravity, structural alterations in the actin and vimentin cytoskeletal architecture could be detected, no significant changes occurred after 11 days microgravity.

### Analysis of metabolites

**Fig 9:**
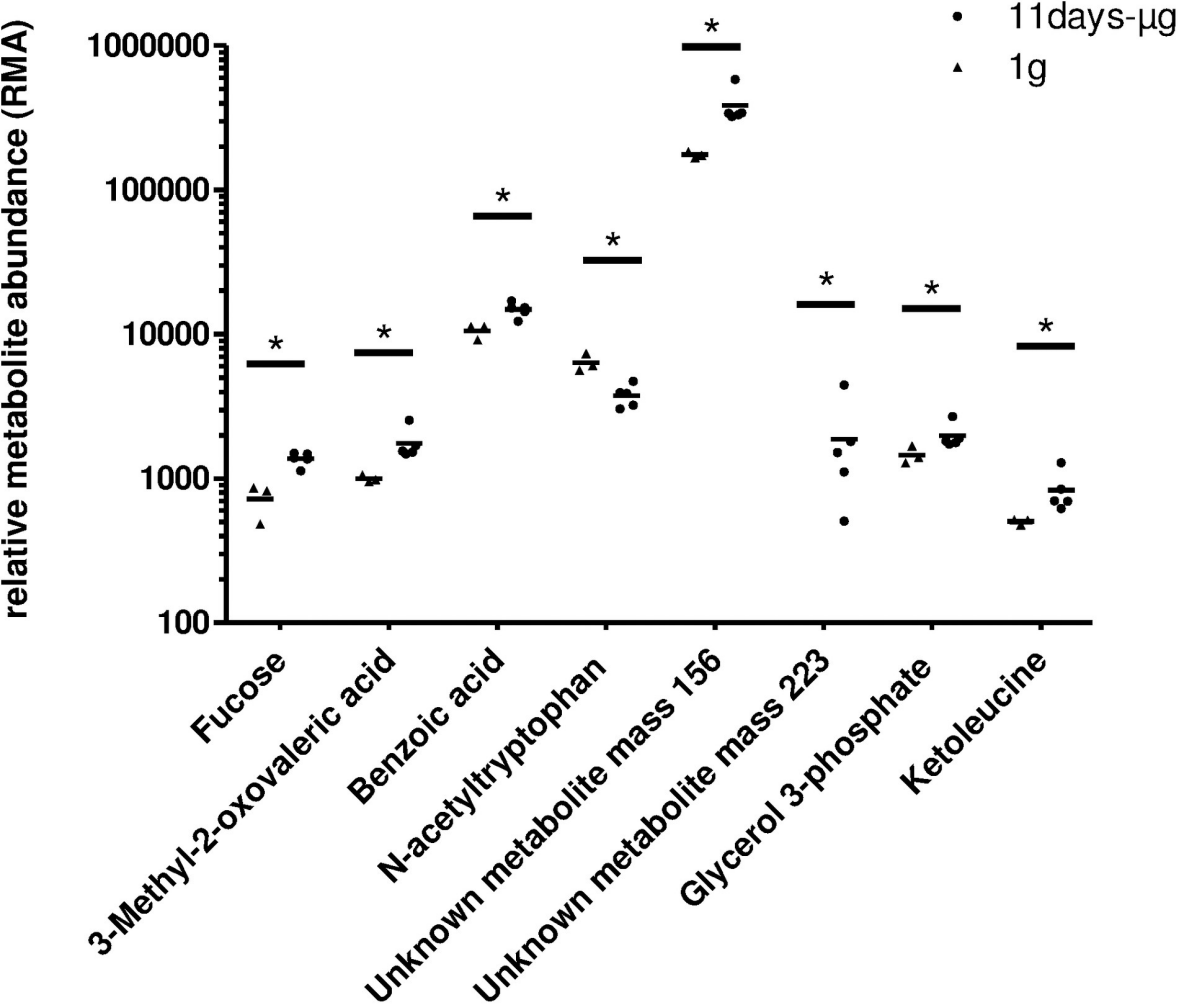
GC–TOF–MS metabolite analysis in cell culture supernatants of primary human macrophages after 11d in microgravity compared to 1g ground controls. Metabolite abundance of 8 significantly changed metabolites are shown, 68 out of 74 analyzed metabolites were not significantly altered. Single data points and means are shown for each experimental group (*p<0.05) and represent values from 3 (1g) or 5 (μg) independent experimental units.

To detect possible changes in the metabolic activity of the cells during different gravitational conditions, the cell culture supernatant was analyzed for a selection of 75 metabolites by GC–TOF–MS. The analysis revealed six known metabolites that showed significantly different quantities when flight to ground supernatants were compared (Fig 9). No significant quantity changes were found in the analyses of the other 69 metabolites (Table 1). While 3-methyl-2-oxovaleric acid (3-methyl-2-pentanoic acid), benzoic acid, glycerol-3-phosphate, ketoleucine (4-methyl-2-pentanoic acid) and fucose were present at significantly higher levels in supernatants of cells on orbit, N-acetyltryptophan was decreased in the flight samples. In the microgravity samples, an unknown metabolite mass of 156 m/z was increased. Interestingly, a high concentration of an unknown metabolite mass of 223 m/z was detected in all microgravity samples. The unknown metabolite mass of 223 m/z was not detected in the 1g control samples.

**Table 1:** GC–TOF–MS metabolite analysis of cell culture supernatants of primary human macrophages after 11d in microgravity compared to 1g ground controls. List of 69 not significantly altered metabolites. 75 metabolites were analyzed.

Alanine
Alanyl-alanine
Arabinonic acid-1,4-lactone
Arabitol
Arginine
Aspartic acid
beta-D-Fructofuranosyl-(2,1)-beta-D-Fructofuranose
Butanoic acid, 2-amino-
Butanoic acid, 3-hydroxy-
Butyric acid, 3-amino-
Citric acid
Cystathionine
Cysteine
Cysteinesulfinic acid
Diisopropanolamine
Dodecanoic acid
Eicosapentaenoic acid
Eriodictyol
Erythronic acid
Ethanolamine
Fructose
Fructose-1-phosphate
Fumaric acid
Galactitol
Glucopyranose
Glucosamine, N-acetyl-
Glucose
Glutamic acid
Glutamine
Glyceric acid
Glycine
Glycolic acid
Hexadecanoic acid, 3-hydroxy-
Hexadecenoic-acid
Homoserine
Inositol, myo-
Isoleucine
Isomaltose
Lactic acid
Leucine
Malic acid
Methionine
N-methyl trans-4-hydroxy-L-proline
Octadecatrienoic acid methylester
Ornithine
Ornithine-1,5-lactam
Oxamide
Pantothenic acid
Phenylalanine (2TMS)
Phosphoric acid
Proline
Propaonic acid, 2-amino-3-ureido-
Psicose
Pyridine, 3-hydroxy-
Pyroglutamic acid
Pyruvic acid
Rhamnose
Serine
Serine, O-acetyl-
Sorbitol-6-phosphate
Succinic acid
Succinic semialdehyde
Sucrose
Tagatose
Threonine
Threose
Trehalose
Tryptophan
Valine

Combined pathway analysis for differently abundant proteins (CD14, ICAM-1) and metabolites (3-methyl-2-oxovaleric acid, benzoic acid, fucose, glycerol-3-phosphate, ketoleucine, N-acetyltryptophan) using IMPaLA (http://impala.molgen.mpg.de; version 9, build January 2015), Reactome (http://www.reactome.org; version V53) and MetaboAnalyst 3.0 (http://mirror.metaboanalyst.ca/MetaboAnalyst; version last modified 2015-07-12) yielded no pathway result comprising of more than two search entries.

### Release of surface-bound fucose after 11 days of microgravity

**Fig 10:**
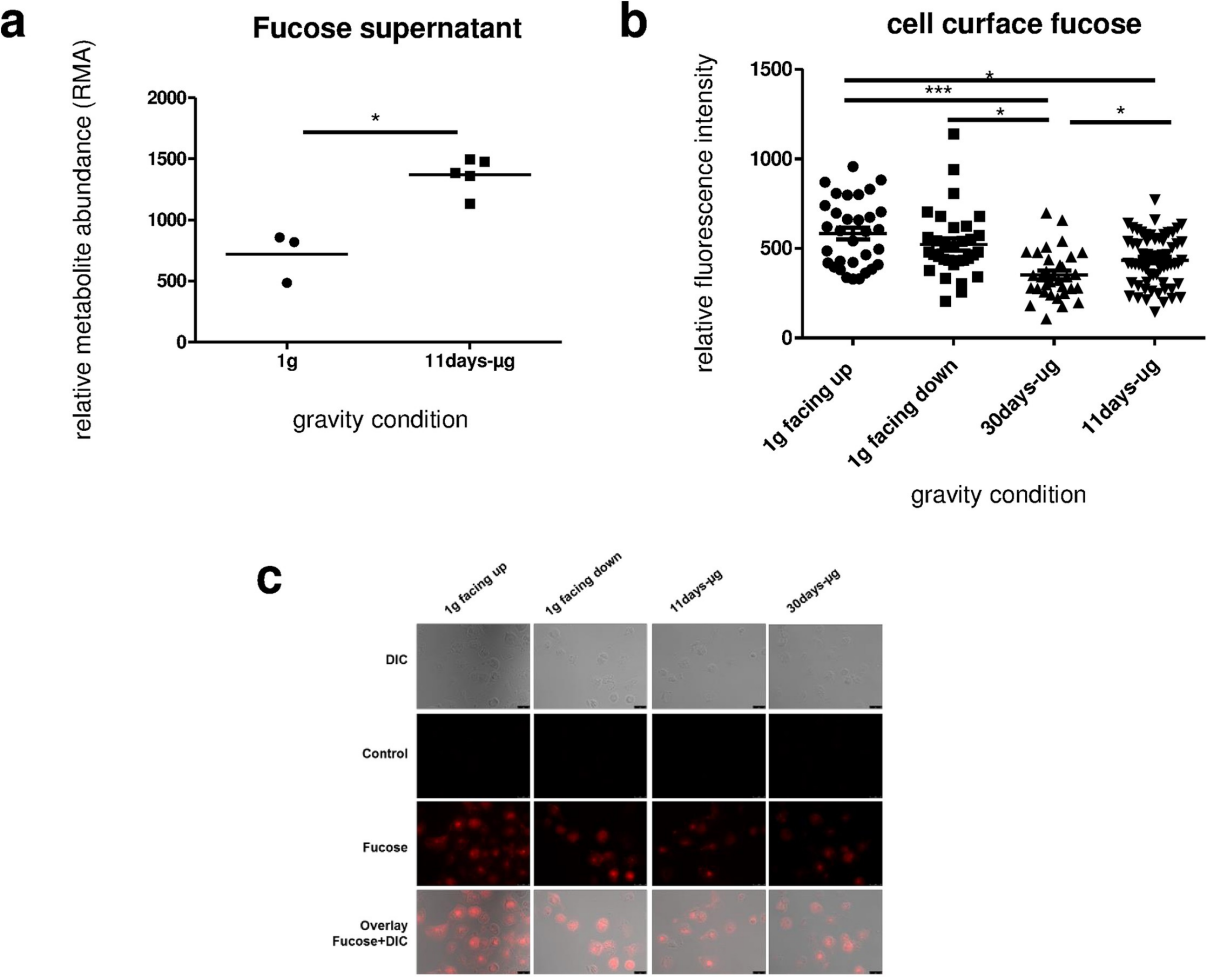
Quantification of fucose in the cell culture supernatant and on the cell surface of primary human macrophages exposed to different gravity conditions. (a) Fucose in the cell culture supernatant was measured by GC–TOF–MS. (b) Surface-bound fucose on cells was measured by fluorescence staining and subsequent quantitative analysis. Data are expressed as relative fluorescent intensity (RFI). Data points represent the mean RFI of all cells on one picture. 32–64 pictures (502–1121 cells) were analyzed per gravity condition. Sample groups 1g facing up (583.7 +/- 33.0, n = 32), 1g facing down (522.3 +/- 33.6, n = 32), 30d-μg (351.9 +/- 24.0, n = 32) and 11d-μg samples (434.0 +/- 17.3, n = 64) are demonstrated. Single data points and means are shown for each experimental group (*p<0.1, **p<0.05, ***p<0.005). **c.** Microscopic images of primary human macrophages exposed to different gravity conditions. Cells stained against fucose (red), differential interference contrast (DIC) pictures, controls (without lectin) and overlay of DIC and fucose are shown. Scale-bar = 25 μm.

After finding an increased amount of fucose in the cell culture supernatant of the microgravity samples (Fig 10A), we hypothesized that this could be attributed to an enhanced release of cell surface-bound fucose. To investigate a potential de-fucosylation of the cell surface, surface-bound fucose was stained with a biotinylated lectin-streptavidin-system. As shown in Fig 10B and 10C, these measurements revealed that the RFI of the fucose staining was 25% lower in the 11d-μg samples (n = 64) compared to the 1g facing up samples (n = 32). The RFI of the 30d-μg group (n = 32) was 39% lower than that RFI of the 1g facing up group (n = 32). Therefore, the significant increase of free fucose in the cell culture supernatant was associated with a significant decrease of cell surface–bound fucose. No significant difference was found between the 1g facing up and the 1g facing down samples.

## Discussion

We found that primary human macrophages differentiated from monocytes exhibited neither quantitative nor structural changes of the actin and vimentin cytoskeleton after 11 days in microgravity compared to 1g ground controls. We also demonstrated a reduced surface expression of ICAM-1 and de-fucosylation of surface proteins and an altered metabolite spectrum. Due to a technical malfunction during the on-orbit experiment, the experiment time was accidentally prolonged, which provided the unexpected chance to analyze changes after 11 and 30 days in microgravity. Subsequent analysis of every single sample using HCS CellMask and TUNEL staining allowed the exclusion of non-viable or damaged cells and demonstrated that all samples were viable at the time point of fixation.

### Quantitative and structural integrity of the cytoskeleton after 11 days in microgravity

In primary human macrophages, neither quantitative nor significant structural changes of the F-actin and vimentin cytoskeleton were found after 11 days in microgravity. This is a surprising and unexpected finding. Many previous reports clearly demonstrated cytoskeletal alterations after much shorter time in microgravity. Cytoskeletal changes in a microgravity environment have been reported in the time frame of seconds in ML-1 human follicular thyroid cancer cells [30] and in EA.hy926 human endothelial cells [31], in the frame of minutes in A431 human epidermoid cancer cells [32], Jurkat T cells [33], FTC-133 cells [34] and in the frame of hours until days in J-111 cells [16,17], MC3T3 osteoblasts [35], Jurkat T cells [36], MCF-7 human breast cancer cells [37], primary rat cardiomyocytes [38] and human MG-63 osteoblast-like cells [39]. These studies observed different cytoskeletal alterations such as clustering and more perinuclear localization of microtubules, perinuclear localization and clustering of intermediate filaments with larger meshes and perinuclear and cortical redistribution of actin fibers, which were reduced in number, length and thickness [40].

### Cytoskeletal alterations in tumor cell and primary cells in microgravity

Most of the previous studies used tumor cells as a model system. Interestingly, no changes of the actin network were registered in an experiment using primary cardiomyocytes [38]. With 4d in microgravity, the latter experiment also represented one of the longest experiment times *in vitro* [38]. When primary cells and tumor cells are compared, differences regarding the cytoskeletal organization, mechanics and regulation should be considered. Alterations in the structure and organization of cytoskeleton manifest, in most cases, in larger deformability of single cells, as it has been reported for various cancers, such those of the bladder, the prostate, the thyroid and the ovaries [41–46]. Furthermore, these changes are commonly related to either a partial loss of actin filaments [47] or a disorganization of microtubules [48]. As an example, in thyroid cell which are frequently used as a model system for microgravity studies [30,49,50], differences in actin organization between malignant and normal cells may be directly contributing to alterations of cell mechanics [46]. In a recent study, mechanical properties of a variety of normal and cancerous cells were directly compared using atomic force microscopy (AFM) and fundamental mechanical differences between single cancer cells compared to their normal counterparts *in vitro* were clearly described [51]. Therefore, the cytoskeletal architecture and the mechanical properties of tumor cells are not too different from the “microgravity” phenotype described in many studies. This also applies particularly for the molecular mechanisms: Regarding cytoskeletal reorganization, RhoGTPases are ideal candidates to explain the structural cellular changes in microgravity [39,52] and a model describing the regulation of RhoA and Rac1 activities in microgravity, was proposed: During short-term exposure to microgravity, RhoA may be inhibited in order to allow cytoskeleton reorganization with respect to the new mechanical status, while Rac1 is activated to control peripheral actin polymerization. However, in prolonged exposure to microgravity, both RhoA and Rac1 may be inhibited [52]. Importantly, in various tumor cells, RhoA and Rac1 expression and/or activity is increased [53]. Rho GTPases are involved in all stages of cancer progression [54] and affect tumor cells by modulation of gene transcription, cell division and survival, intracellular transport of signaling molecules or modification of the interaction of cancer cells with surrounding stromal cells [54]. Besides from contributions to physiological processes, Rho GTPases have been found to assist cancer cell migration, invasion, and metastasis [54,55]. It is therefore possible, that a similar molecular machinery is involved in microgravity-induced cellular response and tumor cell function. Due to the similarity between cytoskeletal alterations in microgravity and in tumor cells regarding the loss of actin filaments [47] and the disorganization of microtubules [48], and due to the potential crucial function of RhoGTPases both in tumor cell biology [53–55] and in microgravity-induced cellular re-organization [52], the question whether tumor cells–also used in our own previous studies [19,56,57]—are a truly suitable model to understand fundamental processes in microgravity, arises. The question must be asked particularly with regards to analyses of changes of the cytoskeleton. The significance of the *in vitro* model system–even when all appropriate control experiments were conducted carefully–should be cautiously evaluated. This is especially true, when general biological questions are addressed, specifically concerning manned spaceflight, exploration and the appropriate risk assessment and monitoring [1–4].

### Limitations of the ISS experiment

Only after 30 days in microgravity, a significant decrease of actin and vimentin quantity and severe structural changes were observed. However, the technical failure of the on-orbit fixation procedure was only discovered after sample recovery. An appropriate parallel control experiment was not conducted for the 30-day timespan. It is therefore impossible to determine the influences of re-entry and landing, as well as those of the prolonged culture time. Additionally, the samples were fixed after landing and recovery, therefore other influencing factors, particularly those due to the re-entry and landing deceleration cannot be excluded. In contrast, the 11d experiments was executed in parallel with identical 1g ground controls using identical hardware and identical processes. In all experiments, dead or damaged cells were excluded from every single analysis. Due to limitations regarding the maximal number of EUEs, primary human M1 macrophages for all experimental groups were differentiated from one donor in order to exclude inter-individual effects and the study design is therefore not allowing to address the population variability.

### Gravity-dependent ICAM-1 regulation

After 11 days of microgravity, we detected a decreased expression of the cell adhesion molecule ICAM-1 in primary human macrophages. ICAM-1 protein and mRNA expression was investigated in cells of the monocyte/macrophage system in microgravity during clinostat, parabolic flight (13th DLR PFC and 19th DLR PFC), sounding rocket (TEXUS-49) and orbital experiments (SIMBOX/SZ-8) [19,56,57]. ICAM-1 expression was increased in macrophageal differentiated human U937 cells during the microgravity phase of parabolic flights and in long-term simulated microgravity provided by a 2D clinostat or during real microgravity on the orbital SIMBOX/Shenzhou-8 mission [56]. In murine BV-2 microglial cells, downregulation of ICAM-1 expression in clinorotation experiments and a rapid and reversible downregulation in the microgravity phase of parabolic flight experiments were detected [56]. Therefore, the effect of microgravity on ICAM-1 in cells of the MMS is not consistent, which could be the consequence of different cell systems (tumor cell models in various states of differentiation versus primary cells) and experiment times. Finally, ICAM-1 may be considered a gravity-responsive molecule in mammalian cells. A reduced surface expression of the adhesion molecule ICAM-1 may result in a disturbed activation of CD4+ T lymphocytes and the specific immune response. Interestingly, ICAM-1 and CD14 expression in the 1g controls was dependent on the direction of the gravity vector, and showed a reduced expression at “facing down” position which is associated with a tractive force at the basal cell surface attached to the polycarbonate slide. We therefore assume an indirect gravity-induced effect initiated by mechano-sensitive signaling at the cell-polycarbonate binding area.

### Metabolic changes

GC–TOF–MS analysis of metabolites in cell culture supernatants revealed 69 unchanged and 6 altered metabolites. Human Metabolome Database (HMDB; http://www.hmdb.ca; version 3.6) was used to identify pathways, in which the differentially regulated metabolites are active [58–60].

After 11d microgravity, 3-methyl-2-oxovaleric acid (3-methyl-2-pentanoic acid), benzoic acid, glycerol-3-phosphate and ketoleucine (4-methyl-2-pentanoic acid) were increased in cell supernatants compared to ground controls. 3-Methyl-2-oxovaleric acid (HMDB00491) and ketoleucine (HMDB00695) are metabolites of isoleucine. 3-Methyl-2-oxovaleric acid is produced from isoleucine by cytosolic branched chain aminotransferase 1 and further degraded by branched chain keto acid dehydrogenase E1 to 2-Methyl-1-hydroxybutyl-ThPP. 3-Methyl-2-oxovaleric acid is used as a marker for a disturbed branched-chain amino acid metabolism [61]. Benzoic acid (HMDB01870) occurs naturally as free- and bound-forms of benzoic acid esters in many plant and animal species. The origin of the benzoic acid in the cell culture system is unknown.

Glycerol-3-phosphate is synthesized from dihydroxyacetone phosphate in the glycolysis pathway, from amino acids and citric acid cycle intermediates in the glyceroneogenesis pathway and from glycerol by glycerol kinase. Glycerol-3-phosphate is important for the shuttling of reducing equivalents from cytosolic NADH into the mitochondrial oxidative phosphorylation pathway to generate ATP [62] and for de novo synthesis of glycerolipids e.g. for cell membranes.

After 11d microgravity, N-acetyltryptophan was decreased in cell supernatants compared to ground controls. N-acetyltryptophan (HMDB13713) is produced almost quantitatively in the process of ascorbic acid-induced release of nitric oxide from N-nitrosated tryptophan derivatives and prevents protein molecules from oxidative degradation by scavenging oxygen dissolved in protein solutions [63,64]. Lower N-acetyltryptophan levels in the supernatants of M1 macrophages in real microgravity compared to M1 macrophages on ground could hint towards a reduced production capability of nitric oxide in the cells on orbit which in turn would lead to a reduced defense against microbial infections.

Interestingly, two unknown metabolites containing the mass fragments of 156 m/z (candidate 156_@RI:1666.32) and 223 m/z (candidate 223_@RI:1783.18) were detected in cell supernatants after 11d microgravity. The metabolite including the fragment of 223 m/z was never detected in any 1g sample, but constantly in each microgravity sample. We analyzed the spectra of both unknown metabolites with respect to functional groups. Candidate 223_@RI:1783.18 is suspected to have alcoholic properties while candidate 156_@RI:1666.32 has a similarity to ribose and 2-oxoproline with additional mass fragments. The structures and identities of the unknown metabolites have not been elucidated yet.

### Shedding of fucose from macrophage surface proteins

Surprisingly, we detected a significant increase of free fucose after 11d microgravity in cell supernatants compared to ground controls. While fucose (HMDB00174) is a hexose monosaccharide that is a commonly found in many N- and O-linked glycans and glycolipids produced by mammalian cell, free fucose is very rare. For instance, the AB0 blood group antigens belong to the best characterized fucosylated glycans in humans and the alpha-1→3 linked core fucose is a suspected carbohydrate antigen for IgE-mediated allergy [65]. Fucose is the only L-configurated sugar synthesized and utilized by mammals, lacks the hydroxyl group on the carbon at the C-6 position usually found in other hexoses. Fucose is metabolized by alpha-fucosidase and is elevated in the serum in breast, ovarian, lung and liver cancer, as well as in diabetes and cardiovascular disease [66,67]. Interestingly, after incubation of human monocyte-derived macrophages with alpha-L-fucosidase, an enzyme cleaving terminal alpha-L-fucose from oligosaccharides, the macrophages were no longer able to respond to lipopolysaccharide (LPS) [68]. In our studies, we found an elevated level of free fucose in the supernatant of monocyte-derived M1 macrophages subjected to real microgravity. As the cell culture medium used does not contain any fucose, we suppose that the fucose must have been released from oligosaccharides from the cell surface. This was indirectly confirmed, by detection of more de-fucosylated cell surface proteins. De-fucosylation would therefore render macrophages unable to respond to LPS stimulation or even other activation upon microbial exposure. In this context, P-selectin glycoprotein ligand (PSGL-1) which displays complex fucosylated O-linked poly-N-acetyllactosamine, is present on macrophages and promotes high affinity binding to P-selectin, important in the primary interaction with endothelial cells and activated platelets [69–71]. Recently it has been reported that terminal fucosylation is a novel hallmark of inflammatory macrophages and that inhibition of fucosylation reshapes the differentiation and functions of M1 macrophages, leading to resolution of inflammation in arthritis [72]. Microgravity-induced shedding of fucose from macrophage proteins could therefore enhance the susceptibility to microbial infections due to an impaired innate immune response.

Current studies provided evidences that tissue-resident macrophages and circulating monocytes should be classified as mononuclear phagocyte lineages that are independently maintained in the steady state [73]. In some inflammatory contexts macrophage accumulation does not depend on monocyte recruitment [74]. These findings imply a possible limitation of model character of the applied primary human macrophage cell culture. Blood-derived mononuclear cells were differentiated *in vitro* into M1 macrophages. It was noted that the cell number of all microgravity samples were up to 35% higher than in 1g controls, indicating faster macrophage proliferation in microgravity, as occurring also under inflammatory conditions [73,74].

In our study, primary human macrophages differentiated from monocytes exhibited neither quantitative nor structural changes of the actin and vimentin cytoskeleton after 11 days in microgravity compared to 1g ground controls. This is in contrast to many previous studies using tumor cells. We demonstrated a reduced surface expression of ICAM-1 and de-fucosylation of surface proteins, which might contribute to functional impairment, e.g. in activation of T cells, migration and the innate immune response. We assume that the surprisingly slight and non-significant cytoskeletal alterations may represent a lower susceptibility of macrophages to microgravity-induced cytoskeletal changes and a stable “steady state” after adaptive processes initiated in the new microgravity environment.

Cellular adaptation to the microgravity environment appears to include very complex changes of cellular and molecular mechanisms. It can only be studied and understood in dynamic measurements. However, dynamic monitoring of cytoskeletal organization during long-term exposure to real microgravity is still missing due to the lack of appropriate on-orbit live imaging facilities. Live imaging experiments on board of the International Space Station could crucially contribute to the understanding of the cytoskeletal dynamics of cellular adaptation to microgravity. The required technology has been recently developed by the German Aerospace Center (DLR), a compact confocal laser spinning disc fluorescence microscope (FLUMIAS), which has been tested successfully during a parabolic flight (24^th^ DLR parabolic flight campaign) and suborbital ballistic rocket flight (TEXUS-52 mission). Due to the utmost importance of the human macrophage system, not only because of the elimination of pathogens, but also due of the clearance of more than 10^9^ apoptotic cells per day [75,76], such studies seem an indispensable prerequisite to understand the homeostasis of the human immune and body systems in microgravity conditions.
